# Evaluating *Enterococcus faecium* 9 N‐2 as a probiotic candidate from traditional village white cheese

**DOI:** 10.1002/fsn3.3878

**Published:** 2023-11-28

**Authors:** Neslihan Dikbaş, Yusuf Can Orman, Şeyma Alım, Sevda Uçar, Ahmet Tülek

**Affiliations:** ^1^ Department of Agricultural Biotechnology, Agricultural Faculty Ataturk University Erzurum Turkey; ^2^ Department of Herbal Production and Technologies, Faculty of Agricultural Sciences and Technology Sivas Science and Technology University Sivas Turkey; ^3^ Department of Bioengineering and Sciences Iğdır University Iğdır Turkey

**Keywords:** *Enterococcus faecium*, lactic acid bacteria, phytase, probiotic properties

## Abstract

In this study, various functional and probiotic attributes of the *Enterococcus faecium* 9 N‐2 strain isolated from village‐style white cheese were characterized, while also assessing its safety. To achieve this, we conducted an in vitro analysis of several key probiotic properties exhibited by the 9 N‐2 strain. Notably, this strain demonstrated robust resilience to low pH, high bile salt concentrations, lysozyme, pepsin, pancreatin, and phenol. Furthermore, this strain displayed exceptional auto‐aggregation capabilities and moderate co‐aggregation tendencies when interacting with *Escherichia coli*. The cell‐free supernatant derived from strain 9 N‐2 exhibited significant antimicrobial activity against the tested pathogens. The strain exhibited resistance to gentamicin, meropenem, and bacitracin, while remaining susceptible to vancomycin and various other antibiotics. Furthermore, it was found that *E. faecium* 9 N‐2 possessed the capacity to produce the phytase enzyme. When all the results of this study are evaluated, it is thought that 9 N‐2 strain has superior probiotic properties, and therefore it can be used as probiotic in food, medicine, and animal feed in the future. In addition, further in vivo tests should be performed to fully understand its effects and mechanisms of action and to confirm its safety and probiotic effects. Further research and clinical trials are also needed to identify new strains with potential probiotic properties.

## Graphical abstract

The isolated strain exhibited good tolerance to low pH, high bile salt, pepsin, pancreatin, lysozyme, and phenol. The 9 N‐2 strain demonstrated excellent auto‐aggregation and moderate co‐aggregation with *E. coli*. The cell‐free supernatant of strain 9 N‐2 exhibited significant antimicrobial activity against a range of tested pathogens. The strain showed resistance to gentamicin, meropenem, and bacitracin and susceptibility to vancomycin and other clinically important antibiotics. We found that the *E. faecium* 9 N‐2 strain has the ability to produce the phytase enzyme.

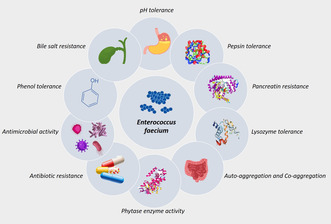

## 1 INTRODUCTION

The term probiotic originates from a Greek word meaning “for life” and is used to describe nonpathogenic living microorganisms that, when consumed in sufficient quantities (10^6^–10^7^ cfu g^−1^), show positive effects on the health of their host with whom they live a symbiotic life (Çam et al., 2023; Salvetti et al., 2016). Scientific evidences show that probiotics have positive effects on the health of the host in terms of regulating microbial balance in the gastrointestinal tract, reducing serum cholesterol levels, alleviating symptoms of lactose intolerance, reducing the risk of colon cancer, increasing the bioavailability of nutrients, preventing harmful substances and pathogens from entering the bloodstream by improving the barrier function of the intestinal lining and improving calcium absorption, and synthesizing short‐chain fatty acids and certain vitamins (Wang et al., 2022; Mazziotta et al., 2023).

Lactic acid bacteria (LAB) are highly favored as probiotic candidates among microorganisms because they have been recognized as safe (GRAS) by FDA (Ben Braiek & Smaoui, 2019). *Enterococcus* belonging to this group are Gram (+), catalase (−), and facultative anaerobic and non‐spore‐forming bacteria (Salek et al., 2023; Zaghloul et al., 2023). Members of this genus are highly tolerant of varying temperatures (10–45°C), pH (4.0–9.6), and salt concentrations (0%–8%). They are widely found on the surface of water, in soil, in some plants, in food (meat products, dairy products, olives, seafood, and vegetables), in the animal intestine, in the human gastrointestinal tract, in the mouth, and in the vaginal cavity (Abril et al., 2022; Dinçer & Kıvanç, 2021; Shi et al., 2020). Since *Enterococcus* contains both pathogenic and commensal microorganisms, its role as different from other LABs continues to be questioned (Hanchi et al., 2018; Yerlikaya & Akbulut, 2020). Although some strains are pathogenic to humans or animals, many strains are nonpathogenic and some are used as probiotics in food, animal feed, and medicine (Ferchichi et al., 2021; Krishna et al., 2022). *E. faecium* is the most researched and preferred species in the *Enterococcus* genus as a probiotic candidate (Shi et al., 2020). In addition to probiotic properties, some strains have been found to produce proteins with antimicrobial activity such as bacteriocins, alleviating symptoms of human and animal intestinal inflammation and thus having potential health‐promoting benefits (AlKalbani et al., 2019; Ghazisaeedi et al., 2022). Some strains such as *E. faecium* M74, *E. faecalis* Symbiflor, and *E. faecium* SF68 have been proven to have probiotic potential and are used as probiotics in both farm animals and humans (Rahmani et al., 2022; Zommiti et al., 2022).

Traditional village‐type white cheese is made by local people in various regions of Turkey using semi‐skimmed milk and/or skimmed cow's milk with rennet casein. It is white or whitish in color and semi‐soft. Sometimes, there are small eyes in the cheese depending on the quality of the milk. During ripening, it is kept in brine (12% sodium chloride) for 30 days. The taste of traditional village cheese tends to be somewhat bland and salty (Bulut‐Solak & Akin, 2019). The main isolates of white cheese are *Lactococcus lactis* subsp. *lactis*, *Lactococcus lactis* subsp. *cremoris*, *Lactobacillus casei* subsp. *casei*, *E. faecalis* var. *liquefaciens*, and *Weissella paramesenteroides*. However, other bacterial isolates belonging to the genus *Lactobacillus*, *Enterococcus*, *Pediococcus*, *Leuconostoc*, and *Weisella* species can also be found (Meral Aktaş & Erdoğan, 2022). Therefore, isolation and characterization of LAB from specific cheeses are essential for the isolation of new probiotic strains.

A successful probiotic strain should be nontoxic or nonpathogenic, resistant to stomach acidity and bile salts, able to settle and multiply in the gastrointestinal tract, increase the digestibility of nutrients, and create positive effects on health (Chouikhi et al., 2022; Somashekaraiah et al., 2019). Candidate bacterial strains should therefore be thoroughly analyzed for safety and probiotic characteristics. In addition, although there is a wide range of commercial probiotic strains available on the market, it is crucial to isolate and characterize strains from diverse sources in order to increase the likelihood of obtaining effective probiotic strains. The purpose of this study is to assess the probiotic potential of an *E. faecium* 9 N‐2 strain isolated from cheese. In this context, some in vitro tests were performed to determine the strain's tolerance to digestive tract conditions, antimicrobial activity, compatibility with epithelial cells, and basic probiotic properties related to safety.

## 2 MATERIALS AND METHODS

### 2.1 Bacterial strains

The *E. faecium* 9 N‐2 strain was isolated from conventional cheese and identified with an accession number of ON287215 at NCBI. It was protected under the code 9 N‐2 in N. DIKBAŞ culture collection located at the Department of Agricultural Biotechnology, Faculty of Agriculture, Atatürk University. Pathogenic bacteria (*Pseudomonas aeruginosa* (RK‐481), *Bacillus subtilis* (RK‐483), *Staphylococcus aureus* (RK‐484), *Salmonella enteritidis* (RK‐485), *Bacillus cereus*, and *E. faecalis* (RK‐487)) used in the antimicrobial activity test were stored in nutrient broth (Difco) containing 15% glycerol at −80°C in the Plant Clinical Laboratory Culture Collection of the Plant Protection Department of Atatürk University, Faculty of Agriculture.

### 2.2 Acid tolerance test

Acid resistance was tested using the method of Shi et al. (2020) with minor modifications. Strain 9 N‐2 (0.5 McFarland adjusted) was added by 1% (v/v) to MRS broth at pH 2, 2.5, 3, and 7.0 as control and incubated at 37°C for a duration of 3 h. Samples were diluted tenfold in PBS solution (0.01 M pH 6.2) and plated on MRS agar medium. After incubation at 37°C for 36 h, colony‐forming units (CFU) and survival rate of the strain were calculated. Bacterial survival rate (%) = (log CFU N_1_/log CFU N_0_) × 100%. N_1_ represents treatment administration and N_0_ represents control.

### 2.3 Bile salt resistance

Bile salt resistance of bacteria was performed according to Hajikhani et al. (2021) with minor modifications. MRS broth to which 1.5%, 1.0%, 0.5%, 0.5%, 0.3%, and 0.0% (w/v) oxgall were added was inoculated with 1% of a 16 h bacterial strain (adjusted to 0.5 McFarland) and incubated at 37°C for 3 h. Samples were diluted tenfold in PBS solution (0.01 M pH 6.2) and plated on MRS agar medium. Following a 36 h incubation period at 37°C, we determined the colony‐forming units (CFU) and assessed the survival rate of the strain.

### 2.4 Pepsin resistance

To evaluate the resistance of the strain to pepsin, PBS buffer was adjusted to pH 3.0 and 2.0, and 3 mg/mL of pepsin was added. PBS buffer was inoculated with 1% of the active culture (adjusted to 0.5 McFarland) and incubated at 37°C for 3 h (Simsek et al., 2022). CFU and strain survival rate were calculated at the beginning, and at the end of 1 and 3 h of incubation.

### 2.5 Pancreatin resistance

To determine the pancreatin resistance of the strain, 1 mg/mL pancreatin was added to PBS buffer with a pH of 8.0. PBS buffer was inoculated with 1% of the active culture (adjusted to 0.5 McFarland) and incubated at 37°C for 4 h (Simsek et al., 2022). CFU at baseline and at 4 h and the survival rate of the strain were determined.

### 2.6 Lysozyme tolerance

To determine the lysozyme tolerance of the strain, 1% of the active culture (adjusted to 0.5 McFarland) was inoculated into 10 mL MRS Broth with 100 mg/L lysozyme and without lysozyme and incubated at 37°C for 90 min (Tarique et al., 2022). CFU at baseline and at 90 min and the survival rate of the strain were determined.

### 2.7 Phenol tolerance

The assessment of *E. faecium* 9 N‐2's phenol tolerance was carried out in accordance with the protocol outlined by Adithi et al. (2022), with slight adjustments. An active culture, adjusted to a 0.5 McFarland standard, was introduced at a 1% inoculation rate into MRS Broth containing 0.3% phenol, followed by a 24 h incubation period at 37°C. Samples were collected both at the beginning and at the end of the 24 h period, and bacterial counts were determined after a series of dilutions.

### 2.8 Evaluation of antibiotic resistance

The antibiotic susceptibilities of the strain to rifampin (5 μg), trimethoprim (5 μg), bacitracin (10 unit), penicillin (10 unit), gentamicin (10 μg), ampicillin (10 μg), piperacillin (100 μg), ceftazidime (10 μg), meropenem (10 μg), erythromycin (15 μg), tetracycline (30 μg), teicoplanin (30 μg), and vancomycin (30 μg) were observed. From the active cultures (adjusted to 0.5 McFarland), 100 μL was transferred to MRS agar and smear seeded. Antibiotic discs were placed sterile in petri dishes containing bacteria and incubated at 37°C for 24 h (Tilwani et al., 2022). The diameter of the zones formed around the disk after incubation was measured millimetrically. Results were evaluated according to CLSI.

### 2.9 Antimicrobial activity against some pathogenic microorganisms

The antimicrobial activity of 9 N‐2 strain against test pathogens (*P. aeruginosa* (RK‐481), *B. subtilis* (RK‐483), *S. aureus* (RK‐484), *S. enteritidis* (RK‐485), *B. cereus*, and *E. faecalis* (RK‐487)) was determined by agar‐well diffusion method according to Prabhurajeshwar and Chandrakanth (2019). Active cultures incubated at 37°C for 24 h were centrifuged at 5000 rpm for 15 min, and the supernatant was sterilized by microfiltration through a filter (0.45 μm). Wells with a diameter of 6 mm were opened with the help of a sterile stick in Mueller Hinton Agar containing pathogenic bacteria, and 100 μL of supernatants were transferred. After 24 h of incubation, the diameter of the zone formed around the well was measured.

### 2.10 Auto‐aggregation and co‐aggregation

The assessment of the isolate's auto‐aggregation capability was conducted following the methodology described by Nami et al. (2019), with slight adjustments. Observations were made at various time intervals (0, 2, and 5 h), and the percentage of auto‐aggregation was calculated using the formula given below:

$$\text{Auto}-\text{aggregation}\;\left(\%\right)=\left(1-\frac{\mathrm{At}}{A0}\right)x100$$

(At: absorbance at time t and A0: absorbance at 0).

Co‐aggregation of strain 9 N‐2 against *E. coli* was determined at 37°C after 4 h incubation by modifying the method used by Yadav et al. (2017). The calculation of co‐aggregation (%) was made by using the following formula:

$$\mathrm{Co}-\text{aggregation}\;\left(\%\right)=\left(\frac{\left(\mathrm{OD}1+\mathrm{OD}2\right)-2\left(\mathrm{OD}3\right)}{\left(\mathrm{OD}1+\mathrm{OD}2\right)}\right)x100$$

OD1, 2, and 3, respectively, represent the optical density of *E. faecium* 9 N‐2, *E. coli*, and the mixture after 4 h.

### 2.11 Phytase enzyme activity

A 0.1 mL aliquot containing phytase was subjected to incubation at 50°C for a duration of 10 min following the addition of 0.25 mL of 2 mM sodium phytate. After the incubation period, the reaction was halted by the addition of 10% (w/v) trichloroacetic acid (TCA). Fifteen min after the reaction, the sample was measured at 700 nm against the blind sample (0.1 mL distilled water +0,25 mL 2 mM sodium phytate + %10 (w/v) TCA) (Demir et al., 2018).

### 2.12 Statistical analysis

All statistical evaluations were made using R software (R Core Team, 2021). For this purpose, the descriptive statistics were computed by using “psych” package (Revelle, 2023). In addition, evaluating the analysis of variance (ANOVA) was made by using “agricolae” package in R software (de Mendiburu, 2023). All experiments were carried out in triplicate.

## 3 RESULT AND DISCUSSION

**FIGURE 1:**
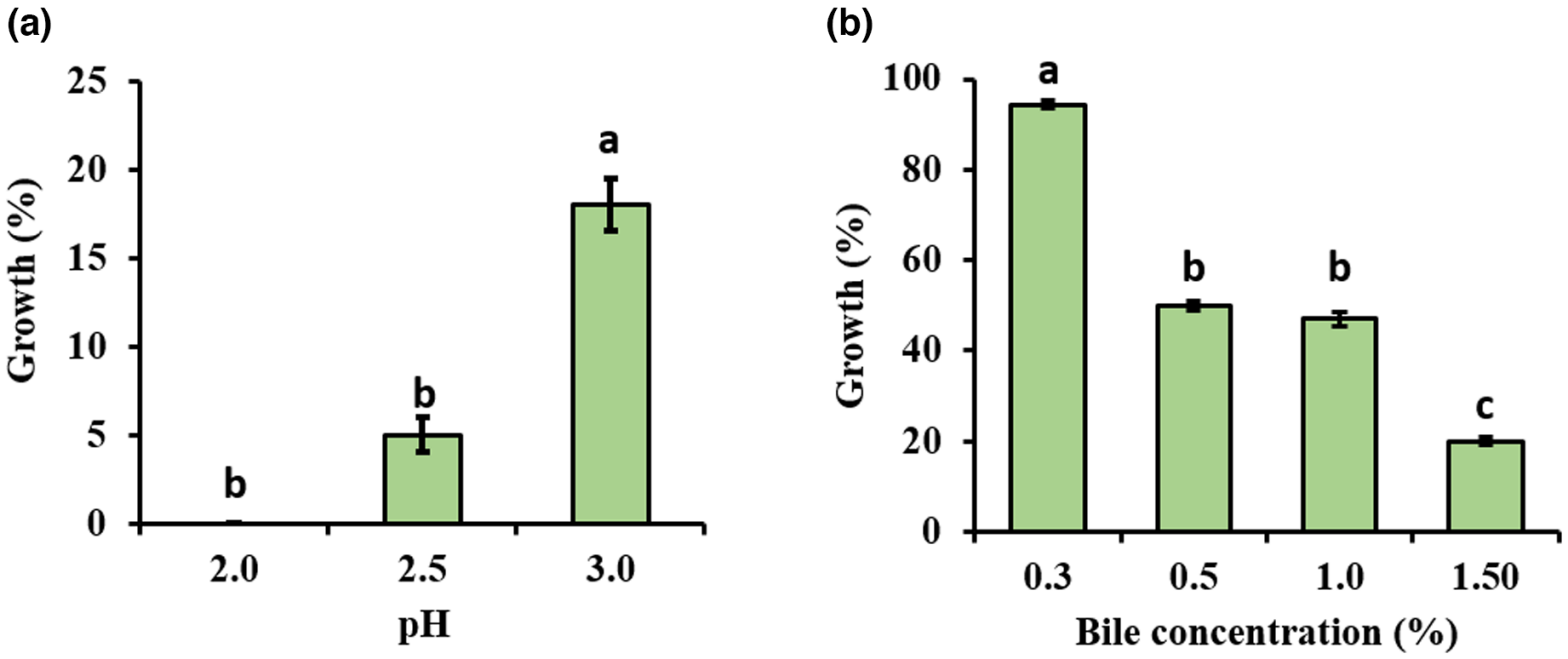
Acid (a) and bile (b) tolerance of *Enterococcus faecium* 9 N‐2. Different letters indicate significant differences (*p* < .05) between treatments according to Duncan's multiple comparison test. All experiments were carried out in triplicate. Bars indicate standard deviation of the mean.

It is extremely important that probiotic strains tolerate the harsh conditions of the gastrointestinal tract (GIT), such as stomach and bile salt acid, before they can exert their proposed benefits in the gut (Alameri et al., 2022). Acid tolerance refers to the ability of probiotic bacteria to survive in an acidic environment such as the stomach, while bile tolerance refers to their metabolic activity in the presence of bile salts in the small intestine and their functional role as probiotics (Ayyash et al., 2021; Ko et al., 2022). *E. faecium* 9 N‐2 was assessed for its ability to tolerate acidity at pH levels of 2.0, 2.5, and 3.0, with its growth under pH 7.0 serving as the control. The pH–growth relationship was analyzed by analysis of variance, and it was determined that the differences between the groups were statistically significant. In addition, the differences were compared by Duncan's multiple comparison test, and the differences between the groups were found to be statistically significant (*p* < .05) (Figure 1a). According to the obtained findings, *E. faecium* 9 N‐2 showed no viability at pH 2.0 after exposure to acidic medium for 3 h, whereas it showed 5% and 18% viability at pH 2.5 and 3, respectively. In this context, it was determined that the pH value at which the strain showed the highest viability was 3. Dinçer and Kıvanç (2021) reported that three *E. faecium* strains (168‐P6, 29‐P2, and 277‐S3) isolated from pastrami could survive for 3 h at pH 2.5. Research indicates that certain types of enterococci strains can endure pH levels of 4.0 and higher. They exhibit limited viability at pH 3.0 and typically cannot survive at pH 2. While it is desirable for probiotic strains to possess acid tolerance within the pH 2.0–4.0 range, a pH tolerance of 3.0 for a duration of 3 h is considered satisfactory to ensure their survival throughout the gastrointestinal system without a significant loss of viability (Dinçer & Kıvanç, 2021; Shi et al., 2020; Tinrat et al., 2018). The obtained results align closely with the existing literature findings regarding the optimal pH range for enterococci.

Bile salts denature surface proteins, disrupting the structure of the bacterial cell membrane and preventing reproduction (Jia et al., 2023). Resistance to bile salts is a crucial factor in the selection of probiotic strains due to the elevated levels of bile salts present in the small intestine and colon. Consequently, the ability of probiotic bacteria to thrive in conditions containing 0.15%–0.30% oxybilene is paramount for their effectiveness (Sharifudin et al., 2021; Wu et al., 2022).

In the study, bile tolerance of the strain was determined after exposure to different bile salt concentrations (0.3%–0.5%, 0.1%, 1.5%) for 3 h. The relationship between bile concentration and growth (%) was analyzed by analysis of variance, and it was determined that the differences between the groups were statistically significant. In addition, the differences were compared by Duncan's multiple comparison test, and the differences between the groups were found to be statistically significant (*p* < .05) (Figure 1b). The *E. faecium* 9 N‐2 strain developed tolerance to various concentrations of bile salt as shown in Figure 1b. After 3 h of exposure to bile, it showed a 94% survival at the lowest bile concentration and a 20% survival at the highest bile concentration of 1.5%. An increase in bile salt concentration was noted to correspond with a reduction in the strain's viability. It was determined that *E. faecium* BS5 strain could tolerate different bile concentrations (2.5%, 5.0%, 7.5%, 10%) for 6 h, and *E. faecium* GMB24 strain could tolerate 0.5%, 1.0%, and 2.0% bile concentrations for 4 h. (BS et al., 2021; Rajput et al., 2022). Similarly, Zaghloul et al. (2023) reported that *E. faecium* EA9 strain could tolerate 0.1% and 0.3% bile concentrations. The 9 N‐2 strain showed a bile tolerance consistent with the literature data.

**FIGURE 2:**
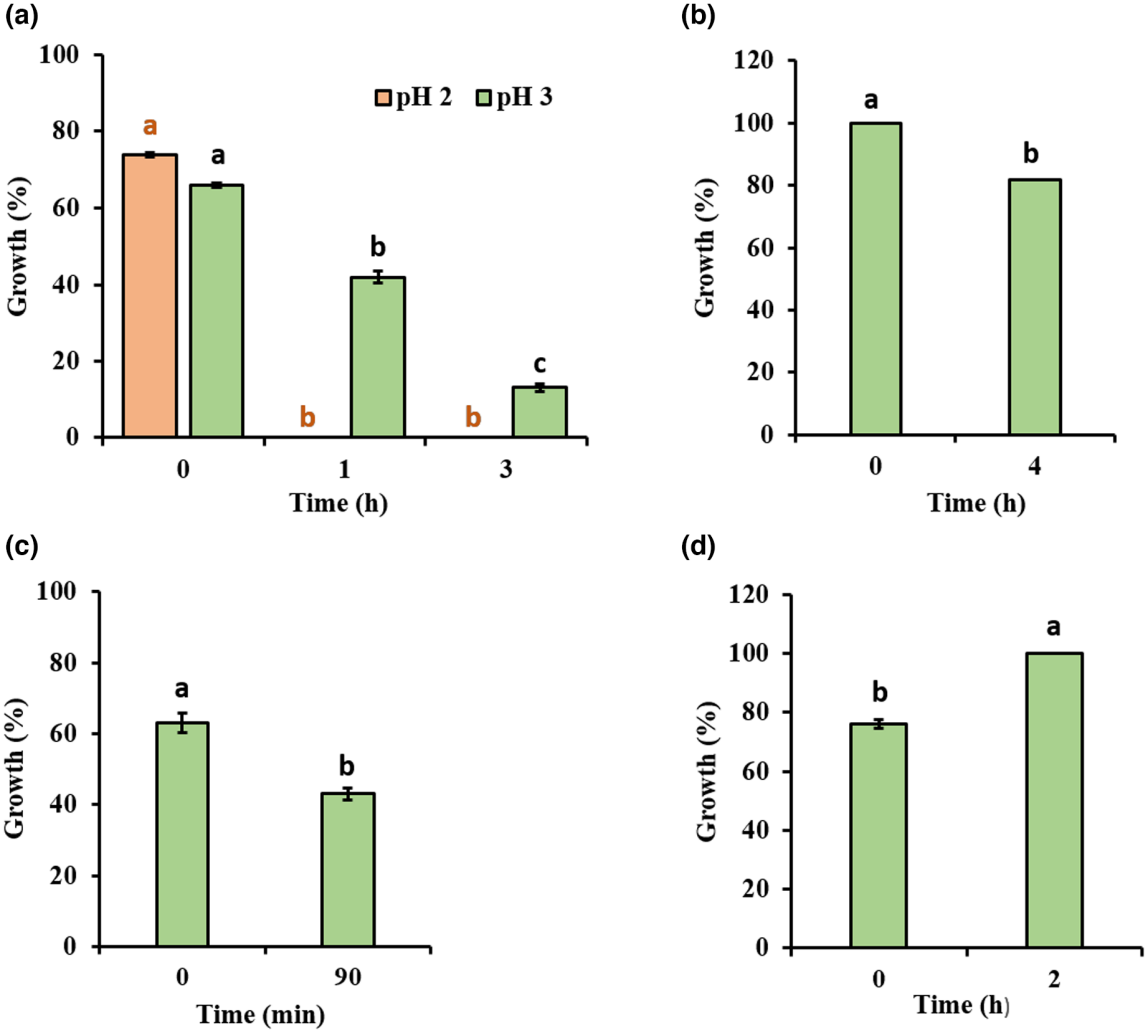
*Enterococcus faecium* 9 N‐2's resistance performance against pepsin (a), pancreatin (b), lysozyme (c), and phenol (d). Different letters indicate significant differences (*p* < .05) between treatments according to independent sample t test. All experiments were carried out in triplicate. Bars indicate standard deviation of the mean.

Tolerance to digestive enzymes is one of the factors to be considered when assessing the potential of a probiotic strain. Pancreatin and pepsin are digestive enzymes produced in the pancreas and stomach, respectively, that help break down proteins into smaller peptides and amino acids (Akmal et al., 2022; Sornsenee et al., 2021). Resistance to these enzymes is desirable in probiotic bacteria as it allows them to survive in the acidic environment of the stomach and the proteolytic environment of the small intestine (Wendel, 2022). To investigate the probiotic power of *E. faecium* 9 N‐2, its resistance to pepsin (at pH 2.0 and 3.0) was tested at three different times (0, 1, and 3 h), and its resistance to pancreatin (at pH 8.0) was tested at two different times (0 and 4 h). The relationship between time–pH for pepsin tolerance of the strain was analyzed by analysis of variance, and it was determined that the differences between the groups were statistically significant. In addition, the differences were compared by Duncan's multiple comparison test, and the differences between the groups were found to be statistically significant (*p* < .05) (Figure 2a). While the isolate did not survive in the presence of pepsin at pH 2.0, it showed a 14% survival rate at pH 3.0 and at the end of the 3 h. Similarly, Singhal et al. (2019) reported that all *E. faecium* strains isolated from the rhizosphere could maintain their viability for 3 h in the presence of 0.3% (w/v) pepsin. In addition, Moussaid et al. (2023) reported that *E. faecium* strains isolated from Moroccan camel milk could survive for 4 h at pH 2.5 in the presence of 3 mg/mL pepsin. The relationship between time–pH for pancreatin tolerance of the strain was evaluated by independent sample t test, and the differences between the groups were found statistically significant (*p* < .05) (Figure 2b). *E. faecium* 9 N‐2 exhibited good resistance to pancreatin with a viability of 82% at the end of the 4 h at pH 8. A similar pancreatin resistance was observed in *E. faecalis* MG5206 and *E. faecium* MG5232 isolated from Kimchi, and both strains survived at pH 7 and 8 in the presence of 1 g/L pancreatin (Kim et al., 2022).

Lysozyme is an enzyme that shows antimicrobial effect by breaking down the cell wall of bacteria (Nawaz et al., 2022). Therefore, fighting lysozyme can be considered a characteristic feature of probiotics. The relationship between time–growth (%) for lysozyme tolerance of the strain was evaluated by independent sample t test, and the differences between the groups were found to be statistically significant (*p* < .05) (Figure 2c). *E. faecium* 9 N‐2 showed 63% and 21% viability after 0 and 90 min of incubation with lysozyme, respectively, and viability decreased proportionally with time. Consistent with the current study, Siddique et al. (2021) stated that *E. faecium* PFS13 and *E. faecium* PFS14 strains, and Tarique et al. (2022) *E. faecium* LB13 strain showed good resistance to lysozyme (100 mg/L) after 90 min.

Phenols synthesized from aromatic endogenous proteins during the digestion process are toxic metabolites (Tilwani et al., 2022). Phenols and their intermediate derivatives can exert bactericidal effects against LAB. Therefore, it is imperative that a given probiotic LAB is tested for their ability to tolerate phenols present in the GIT (de Souza et al., 2019). In this study, the relationship between time–growth (%) for phenol tolerance of strain 9 N‐2 was evaluated by independent sample t test, and the differences between the groups were found statistically significant (*p* < .05) (Figure 2d). We found that the *E. faecium* 9 N‐2 strain was able to tolerate 0.3% phenol after 24 h of incubation at 37°C and remained 100% viable. There was a 21% increase in viability compared to the initial concentration. Similar results for *E. faecium* were observed at different phenol concentrations (0.25%, 0.40%, and 0.50%) for 18–24 h at 30–37°C (Sakkaa et al., 2022; Siddique et al., 2021; Tilwani et al., 2022; Vadassery & Pillai, 2020).

Testing the antibiotic susceptibility of a bacterial strain is an important prerequisite for its safe use in human and animal nutrition (Neut et al., 2017). One of the most important issues in the evaluation of *Enterococcus* as probiotics is antibiotic resistance, especially vancomycin resistance. Foodborne enterococci strains are generally sensitive to clinically relevant antibiotics (ampicillin, vancomycin, and gentamicin) (Abedini et al., 2023; Khan et al., 2022; Krawczyk et al., 2021; Nami et al., 2019). The antibiotic resistance profile of *E. faecium* 9 N‐2 was determined using 13 antibiotics, and the results are presented in Table 1. The isolate was resistant to gentamicin (8.0 ± 0.0 mm), meropenem (10.0 ± 2.0 mm), and bacitracin (12.7 ± 2.5 mm), respectively. Regarding the other antibiotics, it was found to be moderately resistant to teicoplanin (16.7 ± 0.6 mm) and penicillin (18.3 ± 1.5 mm) and susceptible to vancomycin (27.7 ± 1.1 mm) and other antibiotics in Table 1. In the study conducted by Oruc et al. (2021), a similar susceptibility profile against antimicrobials was obtained, and it was determined that nine different *E. faecium* strains isolated from feta cheese were sensitive to tetracycline and vancomycin. In addition, *E. faecium* strains isolated from chicken bile (Shi et al., 2020), poultry intestine (Siddique et al., 2021), and kimchi (Kim et al., 2022) were observed to be sensitive to vancomycin. The *E. faecium* MC‐5 strain has been documented as susceptible to erythromycin, kanamycin, vancomycin, and various other commonly used clinical antibiotics. Conversely, it has exhibited resistance to methicillin, cefazolin, trimethoprim, and nalidixic acid without any alteration in its unique characteristics (Tilwani et al., 2022). Unlike previous studies, 9 N‐2 strain was resistant to gentamicin, meropenem, and bacitracin. Similar results were obtained from *E. faecium* isolates from Tulum cheese, and all of the strains were reported to be gentamicin resistant (Özkan et al., 2021). The 9 N‐2 strain has shown susceptibility to most of the conventional antibiotics that determine its safety and can be applied to foodstuffs, staple commodities, and therapeutic use.

**TABLE 1:** Antibiotic susceptibility of *Enterococcus faecium* 9 N‐2.

Antibiotic	Zone diameter (mm) ± SD*	Antibiotic susceptibility pattern
Ampicillin (10 μg)	22.7 ± 0.6	I
Bacitracin (10 unit)	12.7 ± 2.5	S
Ceftazidime (10 μg)	60.0 ± 0	S
Erythromycin (15 μg)	20.0 ± 0	S
Gentamicin (10 μg)	8.0 ± 0	S
Meropenem (10 μg)	10.0 ± 2	S
Penicillin (10 unit)	18.3 ± 1.5	I
Piperacillin (100 μg)	21 ± 1	S
Rifampin (5 μg)	30.3 ± 0.6	R
Teicoplanin (30 μg)	16.7 ± 0.6	R
Tetracycline (30 μg)	29.7 ± 0.6	S
Trimethoprim (5 μg)	35 ± 3	R
Vancomycin (30 μg)	27.7 ± 1.1	S

Abbreviations: I, intermediate; R, resistant; S, sensitive.

* Values refer to the mean ± standard deviation from a triplicate replicate study.

One of the prerequisites for probiotics is the presence of antimicrobial activity (Silva et al., 2020). Antimicrobial activity is an important requirement as it allows probiotics to suppress pathogen colonization, modulate the immune system, and exhibit anti‐inflammatory activity (Tang & Lu, 2019). In the study, *E. faecium* 9 N‐2 was tested for antimicrobial activity against common enteric pathogens and showed inhibitory activity against all pathogens except *B. cereus* (Table 2). *E. faecium* 9 N‐2 formed a good inhibition zone, especially against *P. aeruginosa*, *B. subtilis*, and *S. enteritidis*. Similar to the current study, *E. faecium* strain isolated from bacon showed antimicrobial activity against important foodborne pathogenic bacteria such as *Listeria monocytogenes*, *B. cereus*, and *S. aureus* (Dinçer & Kıvanç, 2021). In contrast to the results of this study, Ahmadova et al. (2013) reported that *E. faecium* AQ71 isolated from Azerbaijan Motal cheese inhibited the growth of *L. monocytogenes* and *B. cereus* but showed no activity against *Salmonella* and *E. coli* strains.

**TABLE 2:** Antimicrobial activity of *Enterococcus faecium* 9 N‐2 against some pathogens.

Pathogenic microorganism	Well diameter (mm)	*E. faecium*
*Pseudomonas aeruginosa* (RK‐481)	6	+++
*Bacillus subtilis* (RK‐483)	6	+++
*Staphylococcus aureus* (RK‐484)	6	++
*Salmonella enteritidis* (RK‐485)	6	+++
*Bacillus cereus*	6	−
*Enterococcus faecalis* (RK‐487)	6	+++

*Note*: Results are medians of three experiments.

Abbreviations: −, No zone; +, 0–3 mm zone (weak); ++, 3–6 mm zone (good); ++++, Zone diameter greater than 6 mm (strong).

**FIGURE 3:**
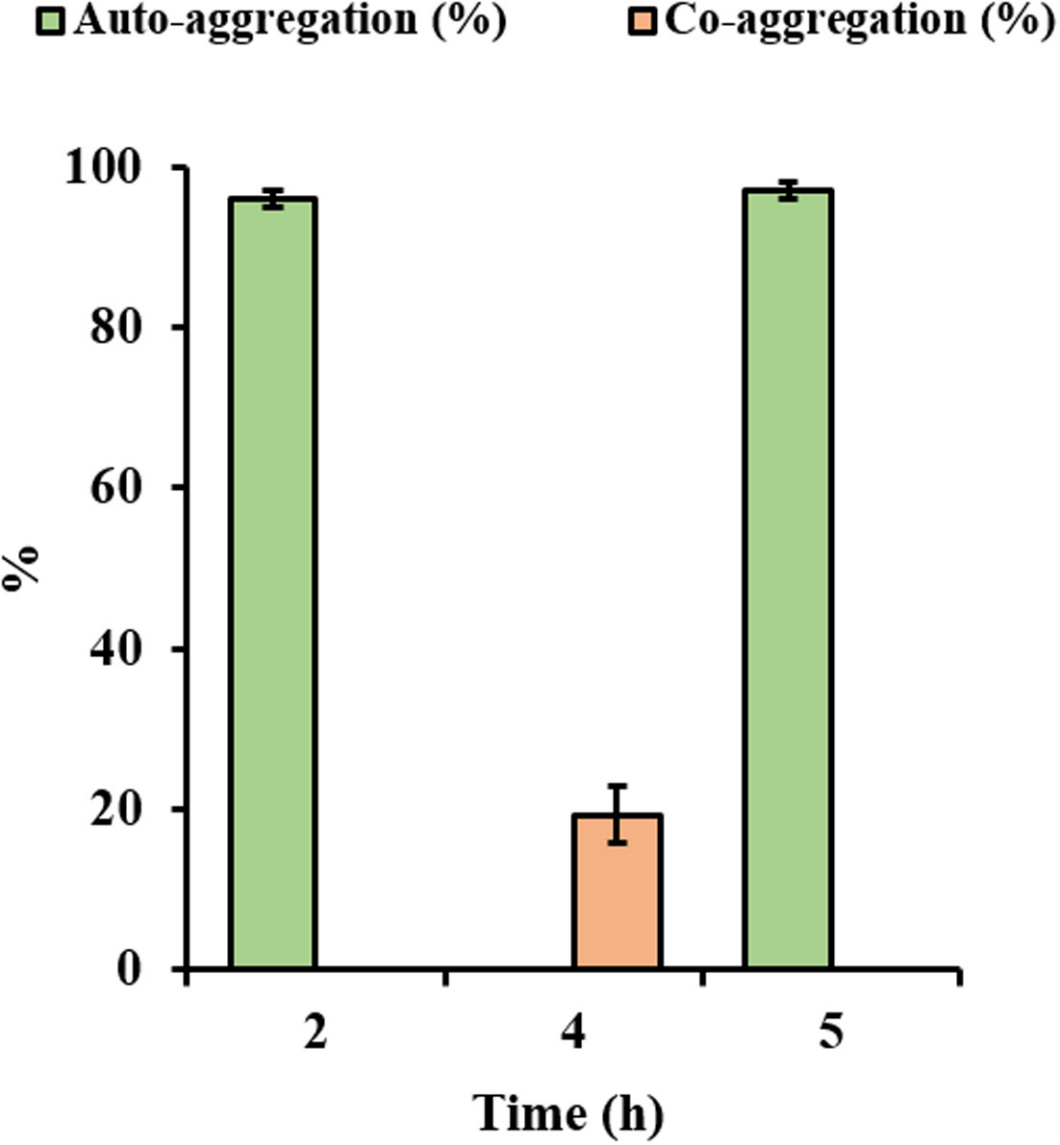
Percentage of auto‐aggregation and co‐aggregation of *Enterococcus faecium* 9 N‐2. All experiments were carried out in triplicate. Bars indicate standard deviation of the mean.

Auto‐aggregation and co‐aggregation are two properties of probiotic bacteria related to their ability to bind to each other and to gut surfaces, preventing biofilm formation and forming clusters that can serve as a first line of defense against pathogens (Nwoko & Okeke, 2021; Tomičić et al., 2022). Auto‐aggregation of probiotic strains is essential for adhesion to intestinal epithelium, and their ability to co‐aggregate can create a protective barrier that hinders the colonization of pathogenic microorganisms. In the present study, the percentage of auto‐aggregation of the strain according to time was evaluated by independent sample t test, and it was determined that the differences between the groups were statistically insignificant (*p* > .05). The proportion of auto‐aggregation of *E. faecium* 9 N‐2 was 95.83% and 97% after 2 and 5 h of incubation, respectively, and the proportion of co‐aggregation with *E. coli* was 19.17% (Figure 3). Zommiti et al. (2018) found that *E. faecium* MZF1‐4, which was isolated from Tunisian meat, exhibited auto‐aggregation ranging from 50% to 96% after 24 h of incubation. In contrast, Vadassery and Pillai (2020) determined that *E. faecium* QQ12, isolated from the gastrointestinal tract of *Oreochromis niloticus*, had an auto‐aggregation proportion of 72.91%. Furthermore, *E. faecium* QQ12 showed a 50.6% co‐aggregation with *A. hydrophila*. Tilwani et al. (2022) determined the auto‐aggregation proportion of *E. faecium* MC‐5 isolated from *Cyprinus carpio* intestine as 100% after 6 h of incubation and reported that the strain showed 100% co‐aggregation with *L. monocytogenes* after 6 h of incubation. The *E. faecium* 9 N‐2 strain showed a higher auto‐aggregation and co‐aggregation compared to the results reported by Zommiti et al. (2018) and Vadassery and Pillai (2020), while it showed similar results to the studies by Tilwani et al. (2022).

Phytate, an antinutrient, binds to essential minerals, reducing their availability for absorption in the human body. Probiotics that produce phytase can degrade phytate by increasing the bioavailability of minerals and combating mineral deficiency in humans. The ability to produce phytase enzyme is a sought‐after trait in probiotics as it allows the breakdown of phytate, improves phosphate bioavailability, and contributes to overall probiotic functionality (Priyodip et al., 2017). Therefore, the ability of the strain to produce phytase enzyme was determined, and the strain exhibited an enzyme activity of 244 U/mL. Similar to the results obtained, there are many studies on LAB phytase production (Demir et al., 2018; Dikbaş, Parlakova Karagöz, et al., 2023; Dikbaş, Uçar, & Alım, 2023; Karagöz et al., 2021).

## 4 CONCLUSION

In the current study, the food‐derived 9 N‐2 strain was examined for safety and probiotic potential. Considering all screening results, *E. faecium* 9 N‐2 appears to be a strain tolerant to low pH, high concentrations of bile salts, digestive enzymes, and antibacterial activity. The strain could be considered a remarkable candidate for future use as a probiotic. However, further in vivo testing should be performed to confirm its safety and probiotic effects before use in food products. In addition, further research and clinical trials are required to verify the probiotics' health claims and identify new strains with potential probiotic properties.

## AUTHOR CONTRIBUTIONS

**Neslihan Dikbaş:** Conceptualization (equal); investigation (equal); methodology (equal); project administration (equal); writing – review and editing (equal). **Yusuf Can Orman:** Conceptualization (equal); investigation (equal); methodology (equal); writing – review and editing (equal). **Şeyma Alım:** Data curation (equal); writing – review and editing (equal). **Sevda Uçar:** Visualization (equal); writing – review and editing (equal). **Ahmet Tülek:** Writing – review and editing (equal).

## FUNDING INFORMATION

This study was supported by Atatürk University Scientific Research Projects (BAP) Coordination Unit (Grant Number: FYL‐2022‐10,137).

## CONFLICT OF INTEREST STATEMENT

The authors have no interests to disclose.

## ETHICAL APPROVAL

No study requiring ethical approval was conducted.

## Data Availability

All analyzed data were included in the current study.
